# Towards generalization for *Caenorhabditis elegans* detection

**DOI:** 10.1016/j.csbj.2023.09.039

**Published:** 2023-10-04

**Authors:** Santiago Escobar-Benavides, Antonio García-Garví, Pablo E. Layana-Castro, Antonio-José Sánchez-Salmerón

**Affiliations:** Instituto de Automática e Informática Industrial, Camino de Vera S/N, Valencia, 46022, Spain

**Keywords:** C. elegans, Detection network, YOLO

## Abstract

The nematode *Caenorhabditis elegans* (*C. elegans*) is of significant interest for research into neurodegenerative diseases, aging, and drug screening. However, conducting these assays manually is a tedious and time-consuming process. This paper proposes a methodology to achieve a generalist C. elegans detection algorithm, as previous work only focused on dataset-specific detection, tailored exclusively to the characteristics and appearance of the images in a given dataset. The main aim of our study is to achieve a solution that allows for robust detection, regardless of the image-capture system used, with the potential to serve as a basis for the automation of numerous assays. These potential applications include worm counting, worm tracking, motion detection and motion characterization. To train this model, a dataset consisting of a wide variety of appearances adopted by *C. elegans* has been curated and dataset augmentation methods have been proposed and evaluated, including synthetic image generation. The results show that the model achieves an average precision of 89.5% for a wide variety of *C. elegans* appearances that were not used during training, thereby validating its generalization capabilities.

## Highlights

• We introduce a detection model trained and evaluated to perform generalist C. elegans detection.
• This model is able to serve as a basis for the automation of numerous assays.
• We evaluate novel dataset augmentation methods.
• These augmentation methods includes synthetic image generation for realistic C. elegans representation.

## Graphical abstract

**Figure:**
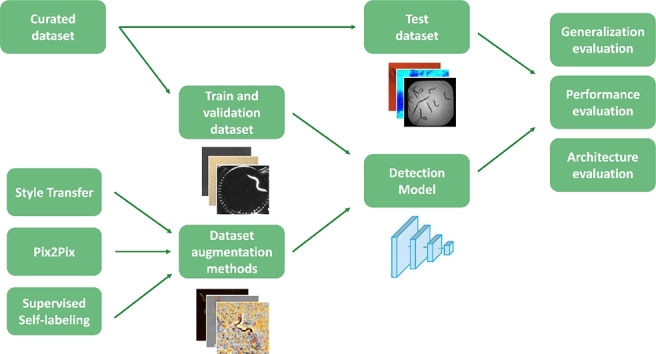

## 1 Introduction

The nematode *Caenorhabditis elegans* (*C. elegans*) is an important model for assays in the field of biomedical research [1]. Its small size, approximately 1 mm, and short life expectancy, between 2 and 3 weeks, are factors that allow for a large number of worms to be easily handled. In addition, the *C. elegans* genome has been fully sequenced and the transparency of its body greatly facilitates observation of both its anatomy and development under the microscope. All these characteristics make it an ideal model for the study of aging and neurodegenerative diseases, as well as useful for the screening of new drugs.

Despite being easy to handle and observe in large numbers, *C. elegans* experimentation entails a high time cost for laboratory technicians performing the tasks necessary to carry out these studies, such as the individual observation of each worm and live-dead classification, for example. This is why it is of great interest to automate these assays, thus freeing the technician from tedious and repetitive tasks, and speeding up the duration of these experiments.

In recent years, various solutions have been proposed to automate assays, such as lifespan [2] [3] or healthspan [4] [5] [6], which are the most widespread assays performed in *C. elegans* to study aging. On developing these solutions, those based on deep learning, using neural networks, currently stand out due to the variety of problems for which they are able to offer effective and robust results. In many cases, when these solutions are implemented, the first step entails the detection of the *C. elegans* individuals themselves.

On the one hand, in object detection-related problems, the currently predominant architectures [7], such as single stage (e.g., YOLO) and two-stage (e.g., Faster R-CNN) convolutional neural networks or transformer-based architectures (e.g., Swin Transformer), offer very good results for this task, as the state of the art has evolved greatly in recent times. On the other hand, observing the implementation of these architectures for *C. elegans* detection, one can see how different solutions have been explored with the Faster R-CNN [8], Mask R-CNN [9] or YOLO [10] models. In all these cases, very good results are achieved, with Average Precision equal to or higher than 0.9, but it is important to highlight how, in all these cases, a specific dataset has been used for training in each case, adjusting the training of these models to a very specific appearance of *C. elegans*.

Generally, when training neural networks for these tasks, pre-trained or randomly initialized models are used as starting points. These pre-trained models use datasets with a large number and variety of images; however, these have nothing to do with the images of nematodes.

We propose a different approach to the ones listed above, working with a model that reaches a good level of Domain Generalization, both for zero-shot inference and for fine-tuning purposes. Although many methods have been proposed for this [11][12], for example self-supervised learning [13] [14] [15] or meta-learning [16] [17] [18], here we mainly focus on dataset augmentation methods.

In this paper, we seek to train a convolutional neural network capable of performing generalist *C. elegans* detection, regardless of the image capture system used and, therefore, of their appearance. In doing so, we aim to achieve a model that has learned a broad representation of the *C. elegans* domain, being able to offer more robust detection. Such a model could be trained with a smaller number of images to reach the desired results, and could therefore be used as a basis for the development of numerous applications for the automation of *C. elegans* assays.

## 2 Materials and methods

### 2.1 Dataset

Since the goal sought is to achieve generalist detection, regardless of the particular appearance of the worms in the image, and as there is no public dataset specifically designed for this, having not been addressed previously, the first step was to create a dataset with a wide variety of different *C. elegans* appearances.

This dataset consists of a collection of public datasets [19] (BBBC011 and BBBC010) [8] [9] [20] and manually labeled images using CVAT. The manually labeled images have been extracted from public videos available on the Internet. These videos have been divided into frames and, in the event of having a good number of frames per second, non-consecutive images have been selected to avoid identical images.

**Fig. 1:**
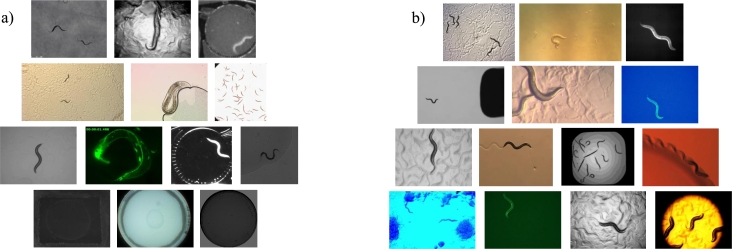
Images used in (a) training, validation and (b) test.

The dataset has been split into 10,612 images for training, 3,773 images for validation (which share the same appearances) and 2,828 images for testing, which consists of 14 worm appearances that are never used in training, in order to evaluate the generalization capacity of the model on new appearances. Altogether, the dataset contains 17,213 images, with 27 appearances from different image capture systems, as can be seen in Fig. 1.

### 2.2 Detection method

The YOLO architecture [21] was chosen to perform the *C. elegans* detection. The YOLOv5s model was used, being one of the fastest of this version. The implementation of (https://github.com/ultralytics/yolov5) has been used.

The fast inference time of this particular model presents a clear advantage over other CNN architectures, allowing its use in real-time applications, without presenting a loss of accuracy in exchange for this feature [22]. This allows the model to be used in a wide variety of applications, both similar to those already explored and validated such as [10] or [23] as well as the integration of this model in hardware systems like [24], allowing real-time tracking of *C. elegans*.

When training this model, we always start from its default weights, pre-trained with the COCO dataset [25], with a batch size=16 for 150 epochs using the SGD optimizer, and a learning rate=0.01 and momentum=0.973.

When training with data augmentation we use, as proposed in the implementation of this model, HSV augmentation (H=0.015, S=0.7, V=0.4), image translation=0.1, image scale=0.5, horizontal flip=0.5 and mosaic probability=1.

This model resizes all images to a fixed size multiple of 32. A size of 1728x1728 pixels has been chosen as it serves as a compromise between the different resolutions, allowing sufficient information to be maintained in macro images, where *C. elegans* have a reduced number of pixels, and allowing for faster training compared to larger dimensions. The hardware used to train the model includes a Ryzen 9 3900X processor with 12 cores running at 3.8 GHz, 128 GB of DDR4 3200 MHz memory, and a Nvidia RTX 3090 GPU with 24 GB of DDR4 memory.

### 2.3 Dataset augmentation methods

Given that we seek to achieve the maximum possible generalization in the detection of *C. elegans*, the ideal situation for training would be to have as many different images as possible with various worm appearances and sources.

There are two main problems, on the one hand, the public availability of *C. elegans* images, and on the other hand, the cost of labeling these images.

First of all, the number of public datasets is not very high, and in some cases, these have not been labeled for either object detection or segmentation tasks. For this reason, we have had to resort to the search for public videos, which have the disadvantage that the frames of the same video offer less variability than the different images in a dataset.

Secondly, the labeling of these images, which is necessary in most cases, can be time-consuming, a fact that also limits the number of images to be added to the dataset.

To try to remedy these problems, a series of methods will be used to augment the dataset, providing more images, and improving the overall variability.

#### 2.3.1 Style transfer

This method aims to create images, with different appearances from those already included in the initial dataset, by transferring image styles that will serve as a source of new appearances to the training images. For this, the AdaIN style transfer model proposed in [26] will be used.

As proposed in [27], this method may allow a convolutional neural network to be less dependent on texture and surface appearance and to focus more on shapes and contours, favoring in this case a more robust detection of the *C. elegans* against variations in their semblance. Also, as can be seen in [28], it has been shown to improve generalization of segmentation in MR images.

**Fig. 2:**
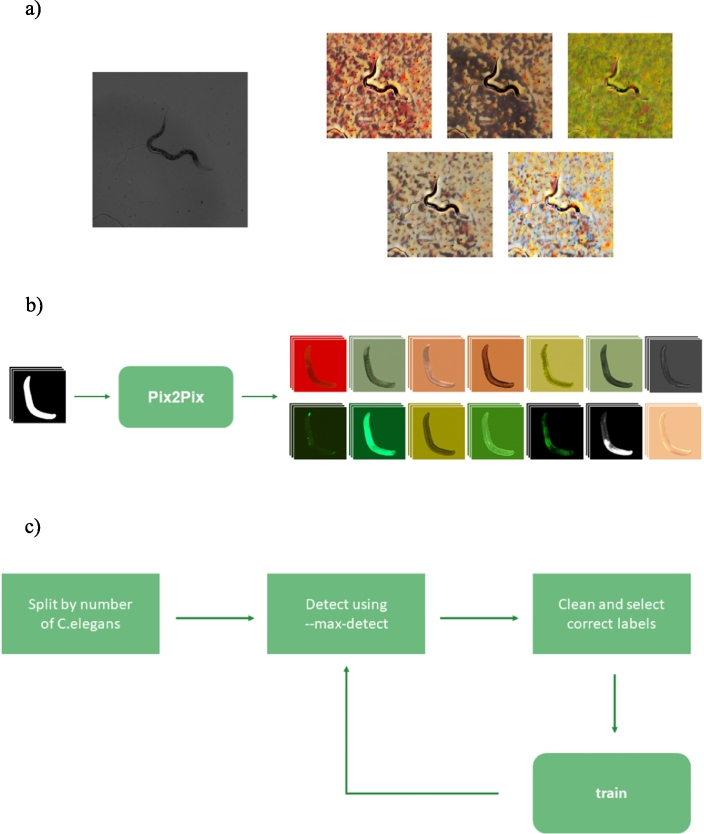
Dataset augmentation methods proposed: (a) Style Transfer, (b) Pix2Pix and (c) Supervised self-labeling.

We used (https://github.com/bethgelab/stylize-datasets) as an implementation of the AdaIN model in PyTorch to perform the style transfer. Five different styles have been applied to each of the training images (Fig. 2), producing more than 53,000 new images.

No significant differences were observed in the training results between using an alpha=1 or an alpha=0.5, a parameter that allows the control, between 0 and 1, of the degree to which the style transfer is applied. This test was performed to check if, in this way, preserving more information in the images where the *C. elegans* have less contrast with the background or where they are very small, better results were obtained.

#### 2.3.2 Pix2Pix

This method seeks to create synthetic images, but, unlike style transfer, being able to provide realistic *C. elegans* appearances, as well as different distributions of these in the image, since an image to which style transfer has been applied has the same number of *C. elegans*, with the same pose and in the same place as the original image.

For this purpose, the Pix2Pix model initially proposed in [29] has been used. This model is a conditional GAN model capable of image-to-image translation between two different domains. In order to generate synthetic images, the problem has been reduced to the generation of individual *C. elegans* from binary masks, thus simplifying the problem that the model must face. In this way, the mask provides the shape and pose of the *C. elegans* and the Pix2Pix network is trained to provide different appearances extracted from real images. Between 10 and 15 mask-image pairs were used to train each appearance, although in cases where very few images of an appearance were available, good results were obtained with as few as five mask-image pairs. The model has been trained to generate *C. elegans* for 14 different appearances, as can be seen in Fig. 2.

**Fig. 3:**
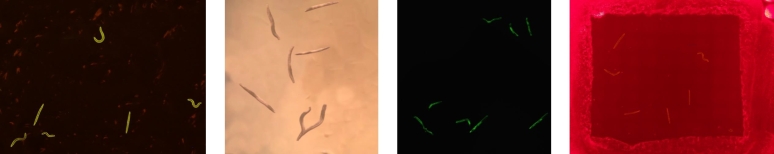
Example of synthetic images generated with the Pix2Pix method.

These individual images are then cropped using the masks that served as an input and randomly assembled into different backgrounds, from which the *C. elegans* have been removed, to generate the final images, as shown in Fig. 3. While being assembled, data augmentation is applied, this is done both to prevent a possible mode collapse in some aspects such as brightness or contrast of the generated *C. elegans*, as well as to increase the variation in the appearance of the images, thus also aiming to favor the generalization capacity of the model. During the process of assembling these images, the labeling corresponding to each one is produced simultaneously, since the placement of each of the *C. elegans* in the background is known. It should be noted that, in this case, only the labeling for object detection, with the bounding boxes corresponding to each image, is generated; however, since we are working with the masks of each of the *C. elegans*, it would be very simple to also generate the labeling for segmentation, assembling the masks in the same position as the *C. elegans*.

**Fig. 4:**
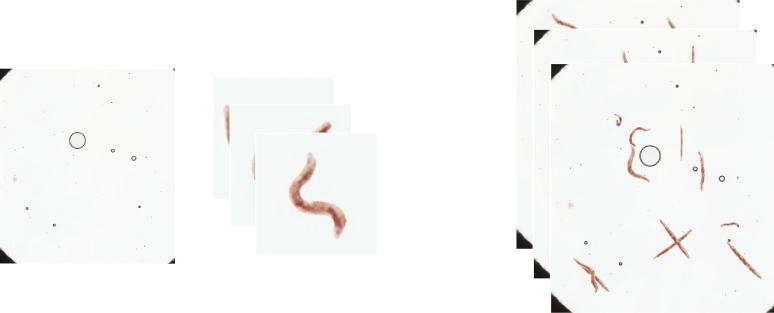
Example of background, *C. elegans* and synthetic images.

To validate this synthetic image generation method, the Pix2Pix model was trained with the appearance of an already labeled dataset (BBBC011). With the generated *C. elegans* and an empty background of this dataset, a dataset with only synthetic images (Fig. 4) was generated.

A YOLOv5s network has been trained on synthetic images only and validated on real images. The model reaches a Precision=0.924, Recall=0.867 and AP@0.5=0.931, thus validating this method of synthetic image generation, capable of matching a given real appearance.

#### 2.3.3 Supervised self-labeling

Unlike the previous methods, this method seeks to provide real images, with appearances that have not been seen before, but using a model formerly trained with the previous methods and which, therefore, already has a good generalization capacity and ability to significantly reduce the time and work required to label new images.

The trained model is used to perform inference on unlabeled images, which are then manually reviewed to incorporate the correct ones in training.

The procedure (Fig. 2) is as follows:

1. All images are separated according to the number of *C. elegans* in each image.
2. Inference is performed using the –*max-detect* parameter to avoid detecting a higher number of *C. elegans* than there actually are.
3. Reading the .txt file of the labels, a Python script is used to delete all the images in which no *C. elegans* have been detected, or the number detected is lower than the real number.
4. Only the images in which the exact number of *C. elegans* has been detected remain. They are manually reviewed and only the correctly labeled images are selected.
5. The model is trained incorporating these images and the process is repeated.

**Fig. 5:**
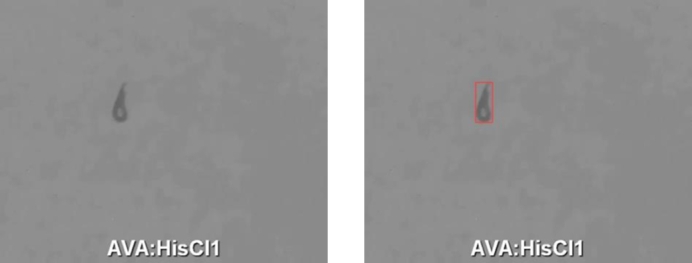
*C. elegans* missed in the first iteration but correctly detected in the following.

Training with the correctly labeled images allows images that were previously missed to be correctly detected in subsequent iterations, as can be seen in Fig. 5.

A total of 6,000 images with 26 completely new appearances were labeled, using this method through three iterations, involving roughly six hours of work. In these 6,000 images there are a total of 10,468 *C. elegans*, assuming that manual labeling can be done at an average speed of 5 seconds per *C. elegans*, it would have taken 14.5 hours to label all the images manually.

### 2.4 Evaluation methods

#### 2.4.1 Training evaluation

To compare the effectiveness of the different augmentation methods, the model is always evaluated against the test dataset having only appearances that the model has never been shown during training, in order to evaluate its generalization capability.

Precision, Recall and AP@0.5 will be used as evaluation metrics.

• **Precision**. It represents the ratio of detections that are true positives to the total number of detections.
  (1) $$Precision=\frac{TP}{TP+FP}$$
• **Recall**. It represents the ratio of model detections that are true positives of the total number of *C. elegans* in the image.
  (2) $$Recall=\frac{TP}{TP+FN}$$
• **AP**. It represents the Average Precision, being the area under the Precision-Recall curve. AP@0.5 will be used, representing the use of an IoU threshold of 0.5 to determine whether a detection is a true positive or false positive.
  (3) $$AP=\int_{0}^{1}P(R)\;dr$$

To properly evaluate the impact of each method, the experiments will be performed incrementally, with the only difference between one training and the next being the incorporation of only one of the dataset augmentation methods.

#### 2.4.2 Performance evaluation

Two experiments were conducted to evaluate the practical performance of the model.

##### Fine-tuning analysis

On the one hand, it may be interesting to use this model as a pre-trained starting point to perform fine-tuning on a particular image capture system to reduce the number of labeled images required.

To check its usefulness in this respect, the model was sequentially trained, several times, on a dataset that it had not been shown before (since it was included in the test dataset) progressively increasing the number of images, starting from a limited number, and measuring the AP@0.5 in each step. This was done both with the model trained with the methods described above and with the default weights of YOLO, in order to make a comparison.

##### Robustness to strain change analysis

On the other hand, it would be interesting to find out if, for a given image capture system, the use of this model can offer greater robustness when different *C. elegans* strains are used, as their appearance may vary.

**Fig. 6:**
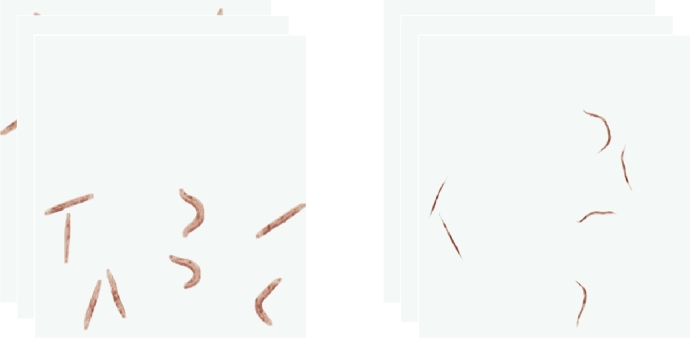
Strains generated using the Pix2Pix model, simulating dpy (left) and lon (right) strains.

To obtain images of *C. elegans* with the same appearance, but pertaining to different strains, two datasets have been generated using the Pix2Pix method, using masks that simulate two notably different strains, but using the same Pix2Pix weights in both cases. These images (Fig. 6) simulate dpy-like (short and wide) and lon-like (long and narrow) strains. The first dataset was used for training and the second one for validation, checking whether there was a drop in the AP@0.5. This was done both for our pre-trained model and for the default weights of YOLO.

#### 2.4.3 Architecture evaluation

We also evaluated the impact that the model used had on the generalization capabilities. To do so, once the best training mode had been determined, after evaluating the proposed methods, YOLOv7 and YOLOv8 models were trained, since these architectures have proven capable of pushing forward the state of the art on the COCO dataset.

The models implemented in (https://github.com/WongKinYiu/yolov7) and (https://github.com/ultralytics/ultralytics) were used. The YOLOv7-tiny, YOLOv8n and YOLOv8s were selected as they are similar in size to the YOLOv5s model. The same hyperparameters were used for training, and the proposed data augmentation parameters of each implementation were used.

### 2.5 Statistical methods

In order to ensure the statistical validity of the results of the above methods, they were repeated three times and the data was evaluated using the Shapiro-Wilk test to check whether the results conform to those of a normal distribution. To do so, the implementation of this test in scipy was used.

Further analysis of the statistical significance of these methods in the results of the experiments were conducted. Depending on the normality of the distributions, given by the Shapiro-Wilk test, this can be done either with the Student's t-test (for normal distributions) or the Wilcoxon signed-rank test (for non-normal distributions).

## 3 Results

### 3.1 Training evaluation

As mentioned above, the model was trained incrementally, changing only one of the dataset augmentation methods at a time. The default weights of the YOLOv5s were always used as the starting point. After the second training, in which the data augmentation techniques described in point 2.2 were included, all the following training experiments included this data augmentation.

The following results displayed in Table 1 were obtained on the validation and test dataset.

**Table 1:** AP@0.5 results after training with the proposed methods: style transfer (ST), Pix2Pix (P2P) and supervised self-labeling (SSL). The experiments are repeated three times on the test dataset and the AP@0.5 values are evaluated with the Shapiro-Wilk test.

	val	test_1	test_2	test_3	p-value
Base dataset	0.938	0.493	0.579	0.508	0.313
BD with data augmentation	0.954	0.693	0.725	0.701	0.463
BD + ST	0.951	0.735	0.742	0.731	0.702
BD + P2P	0.956	0.816	0.810	0.827	0.679
BD + ST + P2P	0.935	0.79	0.795	0.770	0.360
BD + ST + P2P + SSL	0.951	**0.864**	0.857	0.854	0.566

As can be seen in Table 1, the best results were achieved using the three proposed methods, reaching a Precision=0.889, Recall=0.764 and AP@0.5=0.864 on the test dataset.

After evaluating the results with the Shapiro-Wilk test, all the p-values obtained range from 0.3 up to 0.7, therefore the null hypothesis of normality cannot be rejected.

Given that a normal distribution is assumed, a Student's t-test was carried out to test the statistical significance of the proposed methods in the AP@0.5 values. The Style Transfer and the Pix2Pix result values were compared with the results of training with only the base dataset with data augmentation and the Supervised self-labeling result values were compared against the results of training with the Style Transfer and the Pix2Pix methods together.

Table 2 shows the p-values for each method and how each one provides stronger evidence against the null hypothesis than the previous one, being this hypothesis the idea that the methods are not statistically significant in improving the results.

**Table 2:** Results of the Student's t-test for the proposed methods.

	p-value
Style Transfer	0.0544
Pix2Pix	0.0137
Self-labeling	0.0074

### 3.2 Performance evaluation

The training weights obtaining the best results, those of the model trained with the three methods, were selected and the two experiments to evaluate the model performance described previously were carried out.

**Fig. 7:**
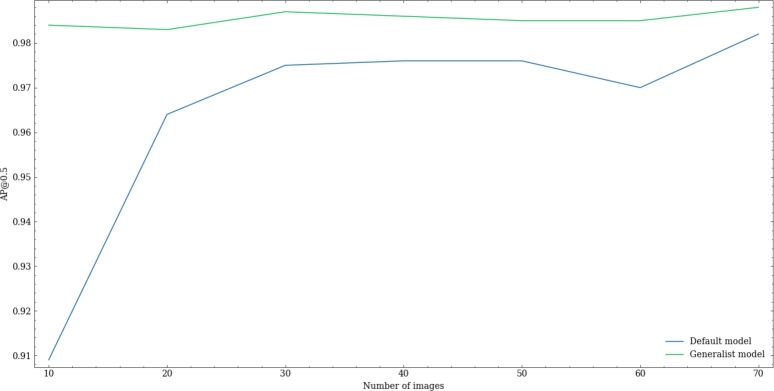
Results of the fine-tuning analysis with the default weights, pre-trained on the COCO dataset, of the YOLOv5s model (blue), and our proposed weights (green).

*Fine-tuning analysis*  On performing the experiment, as shown in Fig. 7, with a much smaller number of images the pre-trained model achieved a higher AP@0.5 than the one obtained after training with the default weights.

*Robustness to strain change analysis*  On performing the experiment, it can be seen how the model trained with the default weights experienced a drop in AP@0.5 of 18% on changing to the validation dataset, whereas when the pre-trained model was used there was only a 2.7% drop, as shown in Table 3.

**Table 3:** Results of the robustness to strain change analysis.

	dpy-like	lon-like
Default model	0.995	0.814
Generalist model	0.995	0.968

### 3.3 Architecture evaluation

Training different YOLO models with the same methods applied before, results in Table 4 show how the YOLOv7-tiny model achieved the highest AP@0.5 on the test dataset, while the YOLOv5s model still achieved the highest AP@0.5 on the validation dataset.

**Table 4:** Results of the architecture evaluation. Different models based on the YOLO architecture with similar sizes have been trained and evaluated both on the validation and test datasets.

	parameters (M)	val AP@0.5	test AP@0.5
YOLOv5s	7.022	**0.951**	0.864
YOLOv7-tiny	6.014	0.939	**0.895**
YOLOv8n	3.005	0.928	0.851
YOLOv8s	11.135	0.944	0.860

## 4 Discussion and conclusions

In view of the results, we can confirm that the methods proposed achieved a satisfactory outcome. The models trained with them provide, on the one hand, better results when working with a reduced dataset, or even reach optimal results with a smaller number of images. On the other hand, they offer great robustness in the event of changes in the appearance of *C. elegans*, such as a change of strain.

In the training evaluation, the YOLOv5s model, for instance, manages to reach values of AP@0.5 up to 0.95 on the validation dataset, while on the test dataset it reaches a maximum of 0.864, by contrast, testing the model on images from a dataset for this type of task (with good lighting, contrast and resolution) like BBBC010, the model reaches a Precision=0.954, Recall=0.921 and AP@0.5=0.964 without prior training for that dataset. Despite this, it is necessary to note that in many cases, when optimal precision is required or when the performance of the model does not reach accuracy requirements, fine-tuning these models to the specific images to be used is recommended, as the model will most likely perform better after this adjustment.

**Fig. 8:**
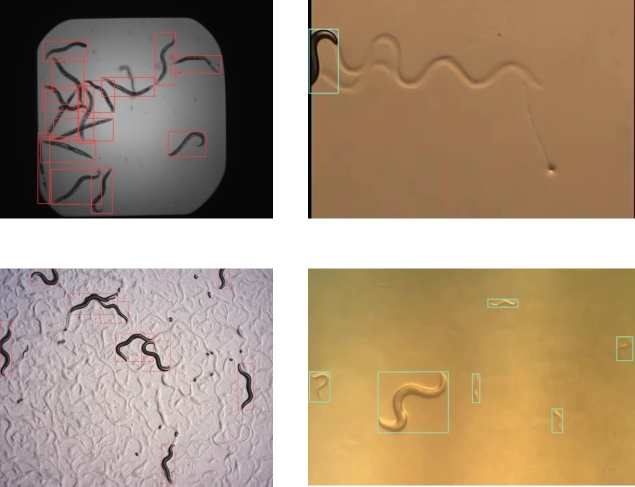
Example of correctly detected *C. elegans* in the test dataset with YOLOv5s (red) and YOLOv7-tiny (blue).

**Fig. 9:**
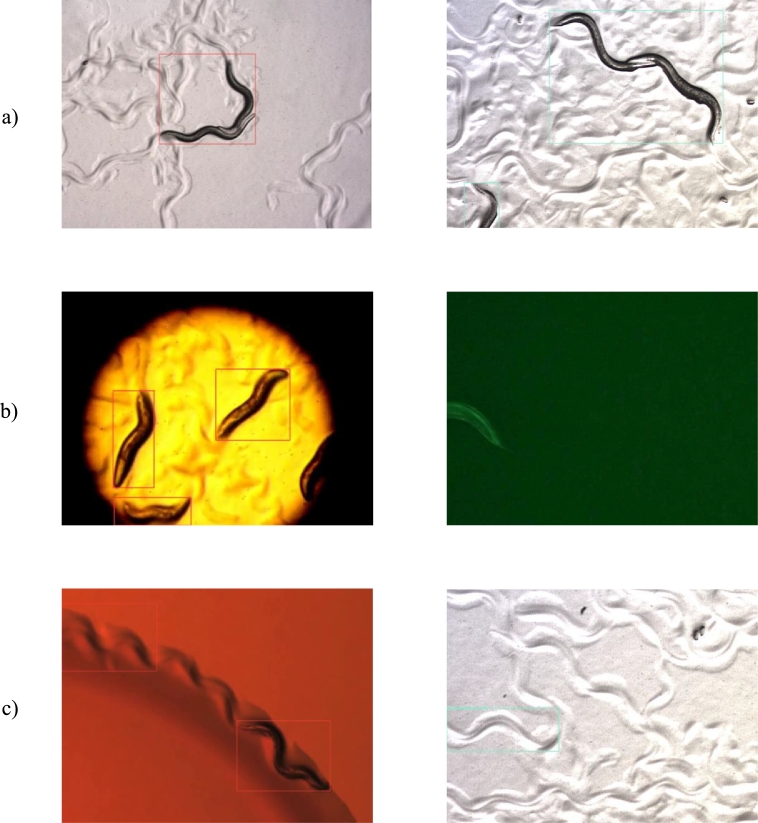
Recurring sources of error observed on the test dataset with YOLOv5s (red) and YOLOv7-tiny (blue), being (a) Aggregation of worms, (b) Partial obstruction of body and (c) Agar patterns resembling *C. elegans*.

Some examples of correct and incorrect detections on the test dataset can be seen in Fig. 8, Fig. 9, respectively. After reviewing these, some recurrent sources of error are (a) aggregation of worms, (b) partial obstruction of the body and (c) agar patterns resembling *C. elegans*. The aggregation of multiple worms is a very well-known problem in *C. elegans* detection, segmentation and tracking, and many solutions have been proposed [30] [10] [31] [32]. Errors (b) and (c), on the other hand, are logical faults of the model and could be reduced with fine-tuning on the target image capture system if this is expected to happen.

In conclusion, we have trained and evaluated several YOLO models to perform generalist *C. elegans* detection, having the potential to serve as a basis for the automation of numerous assays. The best result is achieved by the YOLOv7-tiny model, with Precision=0.830, Recall=0.861 and AP@0.5=0.895 on a test dataset, composed of 2,828 images from 14 image capture systems that are never used in training. Some possible applications of this model include well-known automatic image processing tasks like lifespan, healthspan or tracking [33] [34], but also individual worm detection for posterior analysis such as Life Stage classification [23] or strain identification [35].

With this work we aim to provide a methodology to obtain generic models, based on neural networks, which serve as basic worm-detection models. These models are characterized by performing fully automated worm detection, based on images captured under extremely different conditions, without requiring human input. In this work, we obtain a generic model that serves as a detection model (or as a base model for further refinement or fine-tuning) to achieve end-to-end automation of a wide variety of different assays. With reference to end-to-end automation, this model would enable the localization of all the C. elegans in the image for further processing, as done in [36], for example. It should be highlighted that, depending on the assay, it would be necessary to use specific algorithms or models, as required by the assay, as in [23] or [35], since the detection represents only the first step in image processing.

Also, a new method to generate synthetic images of *C. elegans* using the Pix2Pix model, capable of matching a given real appearance, has been proposed. Further exploration of this method could be interesting for future work, perhaps evaluating its use for segmentation tasks, since this method also allows producing labeled images for segmentation, with their respective masks.

## CRediT authorship contribution statement

**Santiago Escobar-Benavides:** Conceptualization, Data curation, Methodology, Software, Writing – original draft. **Antonio García-Garví:** Conceptualization, Methodology, Software, Visualization. **Pablo E. Layana-Castro:** Conceptualization, Methodology, Software, Visualization. **Antonio-José Sánchez-Salmerón:** Conceptualization, Funding acquisition, Investigation, Methodology, Project administration, Resources, Writing – original draft.

## Declaration of Competing Interest

None Declared.

## Data Availability

We created a repository on github with the weights of our trained models and a list of the original video sources: github.com/SantiagoEscobarBenavides/GeneralistCelegansDetection
